# Modeling Ultrafast Transport of Water Clusters in Carbon Nanotubes

**DOI:** 10.1021/acsomega.3c02632

**Published:** 2023-07-18

**Authors:** Duangkamon Baowan, Ngamta Thamwattana

**Affiliations:** † Department of Mathematics, Faculty of Science, Mahidol University, Rama VI Road, Bangkok 10400, Thailand; ‡ School of Information and Physical Sciences, University of Newcastle, Callaghan, NSW 2308, Australia

## Abstract

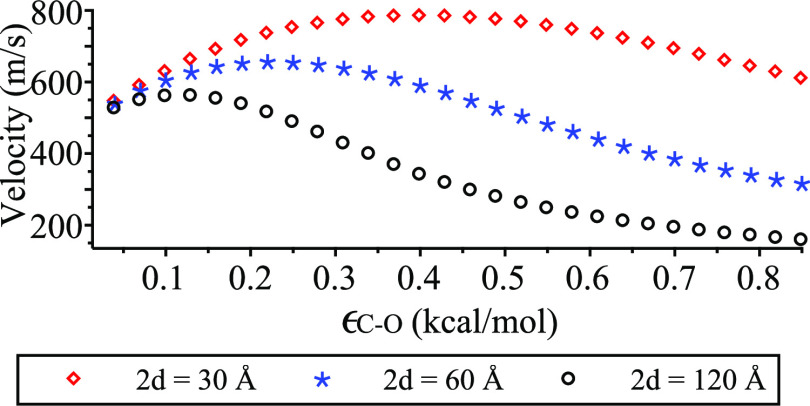

Carbon nanotubes can be used as ultrafast liquid transporters for water purification and drug delivery applications. In this study, we mathematically model the interaction between water clusters and carbon nanotubes using a continuum approach with the Lennard-Jones potential. Since the structure of water clusters depends on the confining material, this paper models the cluster as a cylindrical column of water molecules located inside a carbon nanotube. By assuming the system of two concentric cylinders, we derive analytical expressions for the interaction energy and force, which are used to determine the mechanics and physical parameters that optimize water transport in the nanotubes. Additionally, we adopt Verlet algorithm to investigate the ultrahigh-speed dynamics of water clusters inside carbon nanotubes. For a given carbon nanotube, we find that the cluster’s length and the surface’s wettability are important factors in controlling the dynamics of water transport. Our findings here demonstrate the possibility of using carbon nanotubes as effective nanopumps in water purification and nanomedical devices.

## 1 Introduction

The control of fluid flow in nanoscaled channels is essential to enable the construction of devices used in chip cooling,^1,2^ energy conversion,^3^ water purification,^4−6^ and drug delivery.^7^ To achieve ultrafast speed for liquid transport, carbon nanotubes have been considered as potential transporters.^8−11^ In a review article by Sam et al.,^8^ the water flow slip length inside carbon nanotubes, the physical parameters of the nanotubes, and the interaction constants used in molecular dynamics simulations are shown to be important factors driving the ultrafast transportation of water in the channels. A number of other researchers also study fast transport properties of carbon nanotubes using atomistic approaches, such as molecular dynamics simulations.^9−13^ Their simulations can mimic the flow behavior at very small length and short time scales, which is a challenging procedure for experiments.

Ultrahigh-speed transport can only be achieved by using nanotubes or nanoscaled channels.^14^ In Joseph and Aluru,^9^ various types of nanotubes with radii 10.85–12.20 Å are studied by using molecular dynamics simulations. The flow in a carbon nanotube gives the highest velocity at around 200 m/s, while the velocity of water in a boron nitride nanotube is around 100 m/s. Additionally, the hydrophilic nanotubes, which can be controlled by increasing the interaction strength between carbon and oxygen atoms (ϵ_C–O_), are found to produce lower velocity because the water molecules are more attached to the tube wall. The effect of wettability (hydrophobicity/hydrophilicity) of carbon nanotubes on the velocity of water molecules is also observed by Hummer et al.^12^ In particular, they investigate the interaction between water molecules and carbon nanotubes with ϵ_C–O_ = 0.1143 and 0.065 kcal/mol. They find that more water molecules occupied the space inside the nanotube with ϵ_C–O_ = 0.1143 kcal/mol, which results in the reduction of flow ability compared to the tube with ϵ_C–O_ = 0.065 kcal/mol. Further, Falk et al.^10^ find that the interfacial friction of water on graphitic interfaces is related to the curvature, which depends on the tube’s radius. A decrease in curvature, or an increase in radius, can increase the friction coefficient. This implies that the friction is higher for water on flat graphene or water inside nanotubes with large radii. As a result, Falk et al. observe that water moves faster inside smaller nanotubes. The findings in Falk et al.^10^ and Hummer et al.^12^ are also confirmed by Papadopoulou et al.,^11^ who use molecular dynamics simulations to show that the increase in the tube’s radius and the wettability of the tube’s wall lead to an increase in the friction between the nanotube and the water cluster, resulting in a decrease of the water flow slip length and the flow ability.

While previous studies on fast transport in nanotubes are based on the atomistic computational approach adopting discrete representations of water with various force fields, this paper alternatively employs mathematical principles and techniques to determine the mechanics of transportation of water in a carbon nanotube. In particular, the Lennard-Jones potential and a continuum approach are adopted to determine the interaction energy and force of the system. Having analytical expressions for the total force, Verlet algorithm is employed so that the position and velocity of the water cluster are obtained. Particularly, the effect of both cluster size and wettability on the transport dynamics is studied. We emphasize that the major contribution of this paper is the analytical expressions of the interaction energy and force, which enable us to determine the dynamics behavior of water transport inside a carbon nanotube and the critical parameters impacting the system. Additionally, the formulas presented here are not restricted to only the study of the water–carbon nanotube, but they can be applied to investigate the transport of other types of liquid inside various types of nanotubes.

This paper is structured as follows. In Section 2, the nonbonded interaction function between two atoms is introduced and by using a continuum approach, mathematical expressions describing the interaction energy between two cylindrical structures are derived. Using the interaction energy obtained, the dynamics of water cluster moving in the channel is examined using Verlet algorithm in Section 3. Numerical results and discussion of our findings are presented in Section 4, and finally summary is given in Section 5. Additionally, we confirm the assumption used for modeling a single water molecule as a sphere inside a carbon nanotube in the Appendix of this paper.

## 2 Interaction Energy between Two Nanostructures

### 2.1 Lennard-Jones Potential and Continuum Approach

The nonbonded interaction energy between two atoms at a distance ρ apart can be determined utilizing the Lennard-Jones potential, which is given by
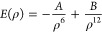
1where *A* and *B* are the attractive and repulsive constants, respectively. For two nonbonded molecular structures, the interaction energy can be evaluated using either a discrete atom–atom formulation or a continuum approach. As mentioned by Girifalco et al.,^15^ the continuum model is closer to reality in order to determine the interaction energy between geometrical nanostructures. Thus, this paper adopts the continuum approximation to examine the interaction between water clusters and carbon nanotubes. In the continuum approach, we assume that atoms are uniformly distributed over the entire surface of each structure, and as a result, the interaction energy is given by
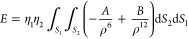
2where η_1_ and η_2_ represent the average surface density of atoms on each molecule. For convenience, we define
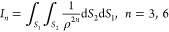
3Thus, eq 2 becomes
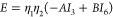
4In terms of the Lennard-Jones constants, the values of well depth ϵ and van der Waals diameter σ are taken from Rappé et al.^16^ Further, the mixing rules^17^ are utilized in the system of two different atomic types, which are  and σ_12_ = (σ_1_ + σ_2_)/2. We note that the attractive and repulsive constants can be determined using the relations *A* = 2ϵσ^6^ and *B* = ϵσ^12^. Here, we consider carbon nanotubes interacting with water molecules (H_2_O), so the constants required are ϵ_C–H_ = 0.06797 kcal/mol, σ_C–H_ = 3.3685 Å, ϵ_C–O_ = 0.07937 kcal/mol, and σ_C–O_ = 3.6755 Å. We note that the wettability inside the nanotubes can be controlled by varying the interaction strength ϵ_C–O_ between carbon and water molecules.^11^ As such, while fixing the values of other constants mentioned here, we vary the value of ϵ_C–O_ in this paper to observe the effect of wettability on the dynamics of transport of water cluster inside a carbon nanotube.

We comment that here we only adopt the Lennard-Jones potential which incorporates both the van der Waals attraction force and Pauli repulsion force to model the interaction between water clusters and carbon nanotubes. For the transport involving charged materials, such as polar-protic molecules within metallic-like carbon nanotubes, the electrostatic potential (the Coulomb potential) must be incorporated together with the dispersion potential to obtain the total energy of the system (see, for example, Thamwattana et al.^18^).

The suction energy proposed by Cox et al.^14^ is the total interaction energy on a molecule entering a channel along the axial direction. For a channel of length 2*L*, the suction energy is given by
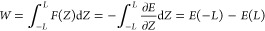
5where *E* is the total energy of the system, which can be obtained from eq 2. Since the dimensions of the water cluster are significantly smaller in comparison to the length of a carbon nanotube, we can take a limit as *L* tends to infinity. As such, we can write the suction energy as *W* = *E*(−∞) – *E*(∞). We comment that the system is stable when the suction energy is positive.

### 2.2 Interaction between Two Cylinders

**Figure 1:**
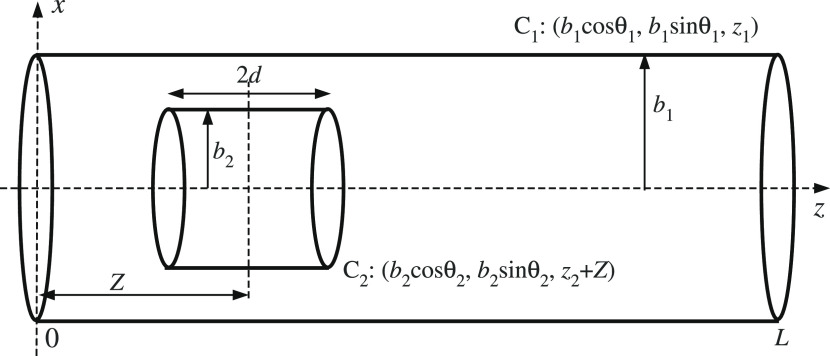
Schematic representation of a water cluster inside a carbon nanotube.

In this paper, we model a cluster of water molecules inside a carbon nanotube as two nested cylinders sharing the same central *z*-axis (Figure 1). The assumption of modeling a water cluster as a nested cylinder is based on the resultant molecular dynamics simulations of Papadopoulou et al.,^11^ which show that the structure of interfacial water depends on the confining material. We note that the details of the lengths and radii of the cylindrical water cluster and the nanotube are given in Section 4.

We refer to the carbon nanotube as the outer cylinder C_1_ and the cluster of water molecules as a cylinder C_2_. The location of the carbon nanotube is fixed while the water cluster is allowed to move along the *z*-axis between the two open ends of the carbon nanotubes, *z* = 0 and *z* = *L*. With reference to the Cartesian coordinates system, a typical point on the cylinder C_1_ of radius *b*_1_ and length *L* has coordinates (*b*_1_ cos θ_1_, *b*_1_ sin θ_1_, *z*_1_), where θ_1_ ∈ [0, 2π) and *z*_1_ ∈ [0, L]. For the cylinder C_2_ of radius *b*_2_ and length 2*d* centered at (0, 0, *Z*), with reference to the same coordinates system, an arbitrary point on the cylinder C_2_ has coordinates (*b*_2_ cos θ_2_, *b*_2_ sin θ_2_, *z*_2_ + *Z*), where θ_2_ ∈ [0, 2π) and *z*_2_ ∈ [−*d*, *d*]. The distance ρ between arbitrary area elements of C_1_ and C_2_ is given by
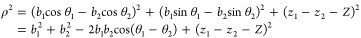
6The integral *I*_*n*_ defined by eq 3 becomes
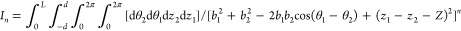
7and we further define
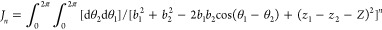
8We use cos2θ = 1 – 2sin^2^θ to obtain
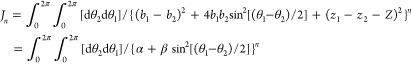
9where α = (*b*_1_ – *b*_2_)^2^ + (*z*_1_ – *z*_2_ – *Z*)^2^ and β = 4*b*_1_*b*_2_. Let
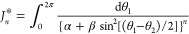
10and it can be shown to be independent of θ_2_ by differentiating *J*_n_^*^ with respect to θ_2_, namely
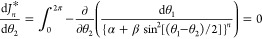
11Thus, we may set θ_2_ to be zero and trivially perform the θ_1_ integration so that eq 8 becomes
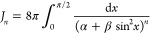
12Following the derivation in Baowan et al.,^19^*J*_*n*_ can be written in terms of a hypergeometric function as
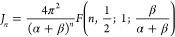
13and the integral *I*_*n*_ defined by eq 7 may be reduced to
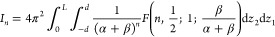
14for *n* = 3 and 6. We proceed to evaluate the remaining integrals in *I*_*n*_ numerically. Thus, the interaction between two co-axial cylinders of water cluster and carbon nanotube is given by
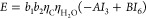
15where η_C_ and η_H_2_O_ are the mean atomic surface densities of carbon nanotube and water cluster, respectively, and *I*_*n*_ is given by eq 14. Lennard-Jones constants,  and , are calculated based on the proportion of hydrogen and oxygen atoms in the water molecules, where *A*_*i*–*j*_ and *B*_*i*–*j*_ are, respectively, the attractive and repulsive constants between atomic types *i* and *j*.

We note that since van der Waals forces are short-range forces, only interactions between nearest atoms need to be considered. As such, we only need to perform the surface integral over the water cluster instead of the volume integral since atoms inside the cluster are farther away from the surface of the carbon nanotube.

Further, we comment that the mean atomic surface density of a carbon nanotube is taken to be η_C_ = 0.3812 atom Å^–2^.^20^ To obtain the mean atomic surface density of a water cluster, we approximate a single water molecule as a sphere with radius equal to the H–O bond length. Thus, the mean atomic surface density of the water cluster can be found from the total number of atoms in one water molecule divided by its surface area, that is, η_*H*_2_*O*_ = 3/[4π(0.9584)^2^] ≈ 0.2599 atom Å^–2^. We comment that the spherical representation for water molecules has been successfully employed in Rahmat et al.^21^ and Tiangtrong et al.^22^ to produce results that are in excellent agreement with molecular dynamics studies. Additionally, to justify this assumption, in the Appendix of this paper, we present the results obtained from modeling a single water molecule as a sphere inside a carbon nanotube, which is shown to be in good agreement with hybrid discrete-continuous model and molecular dynamics simulations.^23,24^

## 3 Dynamics of Particle

Here, we adopt the Verlet algorithm^25^ to monitor the dynamics of a water cluster inside a carbon nanotube. The Verlet algorithm is a numerical regime used in molecular simulation to observe the position and the velocity of an object, namely
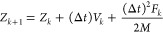
16
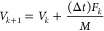
17where *Z*, *V*, *k*, Δ*t*, *M*, and *F* are, respectively, the position of the particle, velocity of the particle, epoch time, time step size, mass of the particle, and total force from the interaction energy. We note that the acceleration in eqs 16 and 17 has been replaced by the total force per mass.

The total force is obtained from *F* = −∂*E*/∂*Z* + *F*_*f*_, where *E* is given by eq 15 and *F*_*f*_ denotes a frictional force. The friction force in the liquid–solid interface is defined by Papadopoulou et al.,^11^ and it can be written as
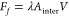
18where *A*_inter_ = 4π *b*_2_*d* is the contact area between the water cluster and the channel, *V* denotes the velocity of the cluster, and λ is the friction coefficient, which depends on the radius of the water cluster and the interaction strength ϵ_C–O_. Here, we adopt the formula given in Papadopoulou et al.^11^ where
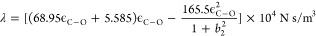
19We note that in the electromagnetism with a dielectric medium, the friction coefficient λ has to be modified to include the electrical force acting on the ions.

In terms of units, we use angstrom (Å) for the unit of the position of water clusters and angstrom per femtosecond (Å/fs) for the unit of the cluster’s velocity. Further, we convert the force unit of 1 (kcal/mol)/Å to 6.9477 × 10^–11^ N, and the density of a water cluster is taken to be 1 g/cm^3^, which is a commonly used measurement for water’s density.^26^ The mass varies depending on the assumed length of the water cluster.

## 4 Numerical Results

Here, the radius of the outer cylinder *b*_1_ is fixed to be 13.56 Å (approximately the radius of a (20, 20) carbon nanotube), and its length is assumed to be 500 Å. In the following two subsections, we vary the length of the water clusters and the interaction strength ϵ_C–O_, which controls the system’s wettability, to investigate their effects on the interaction energy and the movement of water clusters inside the nanotube.

### 4.1 Interaction Energy

**Figure 2:**
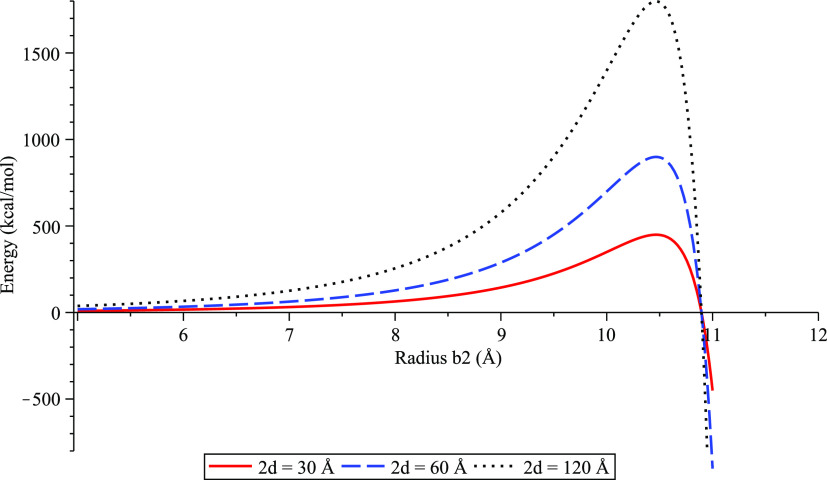
Suction energy eq 5 of water cluster–carbon nanotube interaction for ϵ_C–O_ = 0.040 kcal/mol. We fix the radius of the carbon nanotube to be *b*_1_ = 13.56 Å while vary the lengths of water clusters such that 2*d* = 30, 60, and 120 Å.

The suction energy profiles are shown in Figure 2 for water clusters of three lengths, namely, 2*d* = 30, 60, and 120 Å and ϵ_C–O_ = 0.040 kcal/mol. We note that while the water cluster length *d* can be arbitrary, these three values are chosen based on molecular dynamics simulations shown in Papadopoulou et al.^11^ to study how the variation of the cluster length affects the dynamics behavior of the system. The radius *b*_2_^°^ of the water cluster at *W* = 0 indicates the largest cluster radius that can be encapsulated inside the nanotube of radius *b*_1_. The water clusters with radii greater than *b*_2_^°^ will not be able to enter the carbon nanotube. The radius *b*_2_^*^ of the water cluster at *W* = *W*_max_ maximizes the total energy, hence will lead to the maximum velocity of the water cluster inside the nanotube of radius *b*_1_. We observe that the cluster radii *b*_2_^°^ and *b*_2_^*^ do not change with the lengths of water clusters.

Next, we examine the effect of ϵ_C–O_ on the interaction energy between water clusters and carbon nanotube. We note that low values of ϵ_C–O_ correspond to hydrophobic channel while larger values of ϵ_C–O_ correspond to hydrophilic channel. We obtain *b*_2_^°^ = 10.82–10.90 Å and *b*_2_^*^ = 10.38–10.47 Å when ϵ_C–O_ is in the range of 0.04–0.16 kcal/mol. Further, we define δ_0_ = *b*_1_ – *b*_2_^°^ and δ_*max*_ = *b*_1_ – *b*_2_^*^, which represent the intermolecular distance between a water cluster and the inner surface of a carbon nanotube. We find that δ_0_ ≈ 2.7 Å and δ_max_ ≈ 3.1 Å regardless of the radii and the lengths of water clusters and carbon nanotubes.

**Figure 3:**
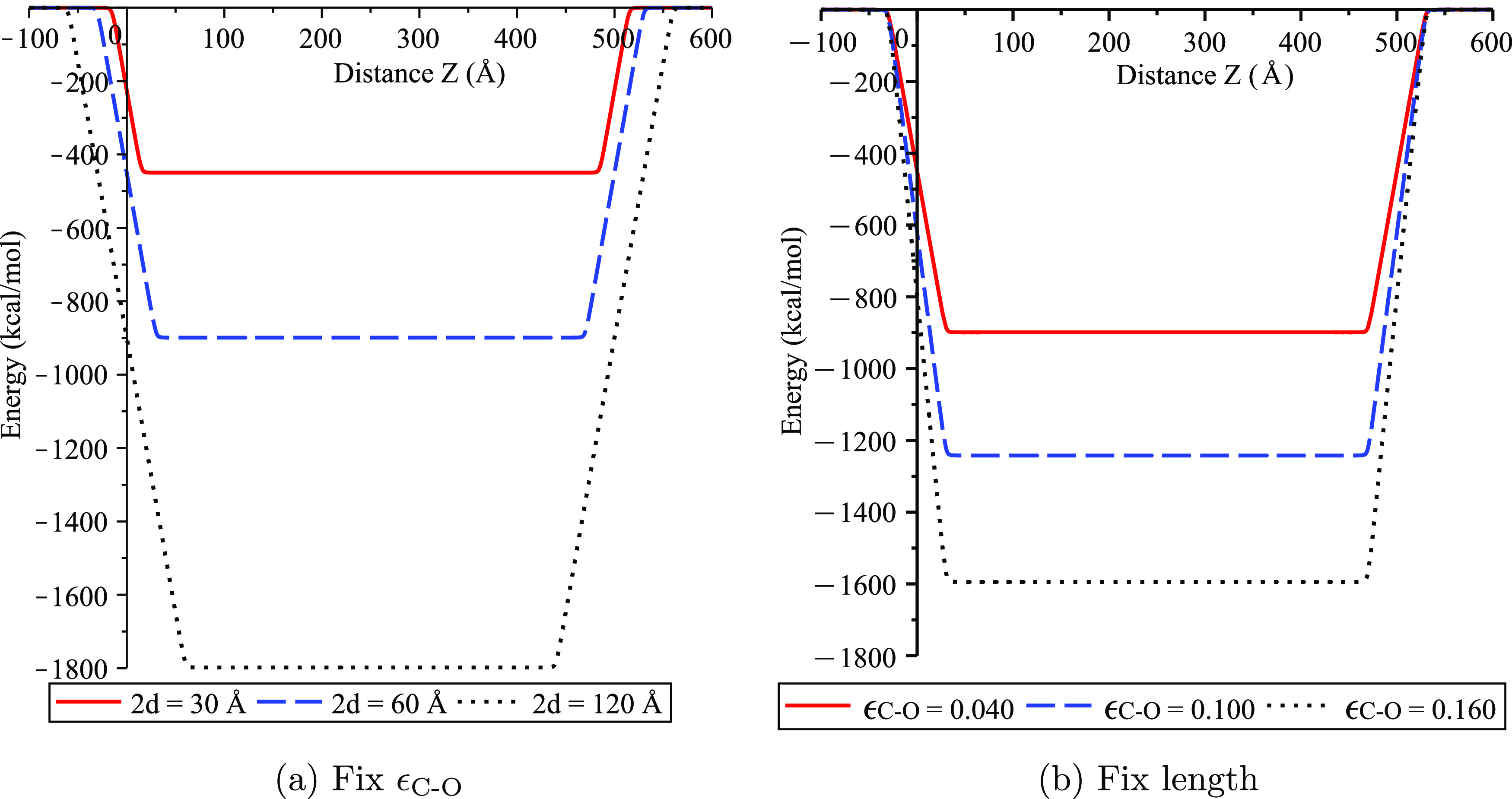
Energy profiles for water clusters in carbon nanotube with *L* = 500 Å and *b*_1_ = 13.56 Å, where (a) lengths of clusters are varied with fixed *b*_2_ = 10.466 Å and ϵ_C–O_ = 0.040 kcal/mol and (b) values of ϵ_C–O_ are varied with *b*_2_ = *b*_2_^*^ = 10.40 Å and 2*d* = 60 Å.

Next, the profiles of the total energy eq 15 are shown in Figure 3 to demonstrate the effects of varying the length of water clusters (2*d*) and the interaction strength (ϵ_C–O_). For the interaction strength ϵ_C–O_ = 0.040 kcal/mol with the cluster’s radius *b*_2_ = 10.466 Å, we find from Figure 3a that higher interaction energy is obtained for the clusters with longer lengths, giving rise to lower energy levels. This is as expected since there are more interacting atoms in the cluster with longer lengths. Similarly, the interaction strength also affects the total energy of the system, as illustrated in Figure 3b, the higher the interaction strength, the stronger the interaction, hence the lower the energy.

**Figure 4:**
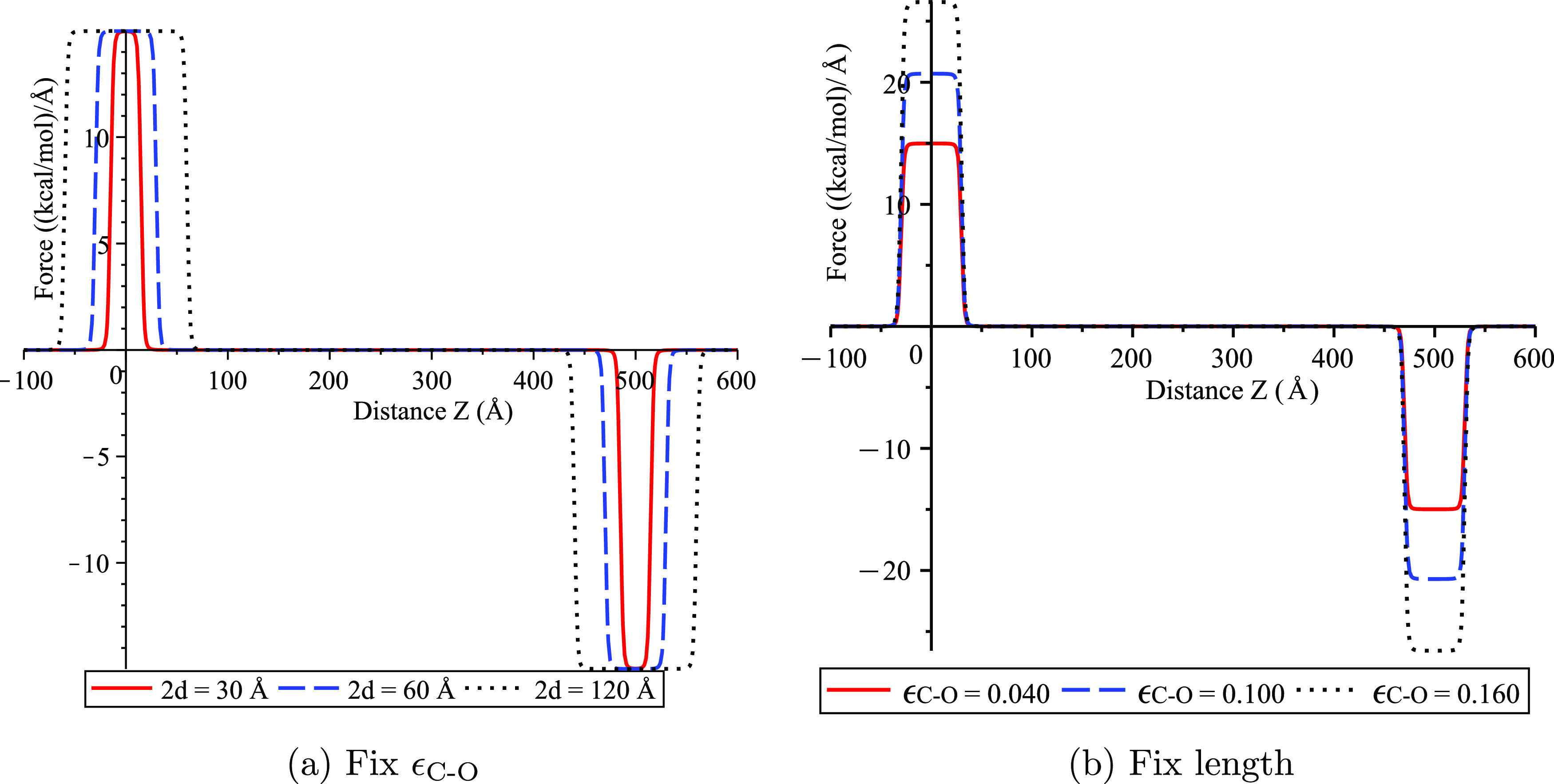
Axial force profiles for water cluster in carbon nanotube with *L* = 500 Å and *b*_1_ = 13.56 Å, where (a) lengths of clusters are varied with fixed *b*_2_ = 10.466 Å and ϵ_C–O_ = 0.040 kcal/mol and (b) values of ϵ_C–O_ are varied with *b*_2_ = *b*_2_^*^ = 10.40 Å and 2*d* = 60 Å.

Force distributions that correspond to the energy profiles shown in Figure 3 are illustrated in Figure 4. We note that only the axial force is considered here due to the symmetry assumed for the two nested cylinders of a water cluster inside a nanotube. The axial force is defined by *F*_*z*_(*Z*) = −∂*E*/∂*Z*, and we observe that the force distributions are also affected by the cluster’s length and the interaction strength. We comment that in Figure 4, the force is ∼0 everywhere except at both ends of the nanotube, where there is a force that attracts the water cluster back toward the center of the nanotube. Since there are no dissipative forces (e.g., frictional force) considered here, the forces at each end of the nanotube operate to generate the oscillation of the water cluster inside the carbon nanotube.

From Figure 4, we can estimate the axial force *F*_*z*_(*Z*) using the Heaviside step function *H*(*x*) as
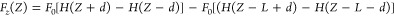
20where *L* is the length of the carbon nanotube, *d* denotes the half-length of the water cluster, and *F*_0_ is the strength of the force at *Z* = 0 and *Z* = *L*. This axial force function together with the frictional force defined by eq 18 is used as the total force *F* in the following subsection to determine the velocity of the water cluster.

### 4.2 Dynamics Behavior

**Figure 5:**
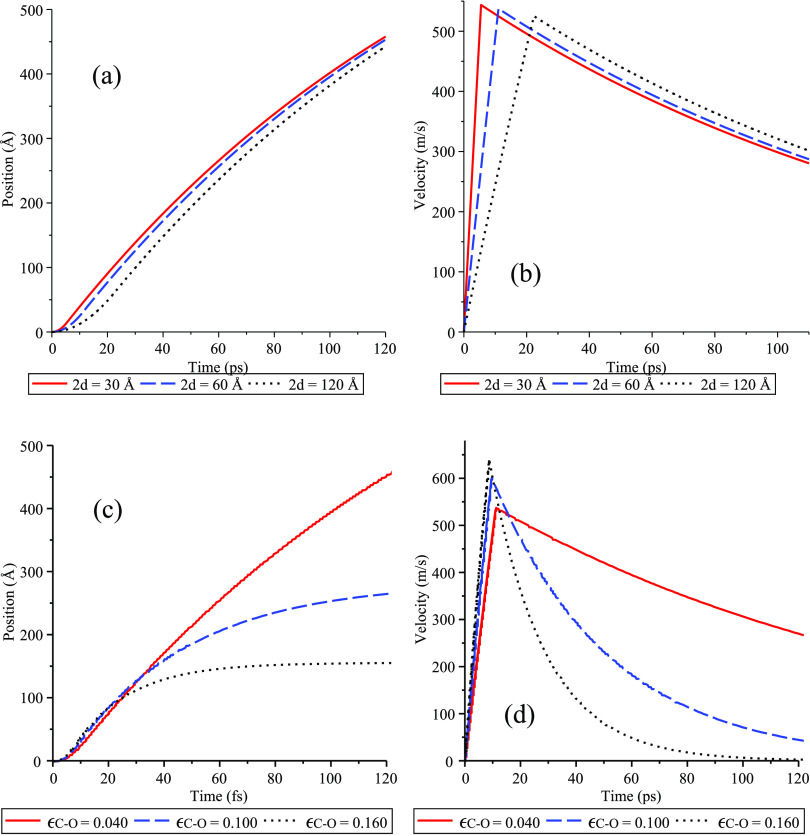
(Left) Position and (right) velocity versus time in ps for water cluster in carbon nanotube of *b*_1_ = 13.56 Å, where (a, b) the interaction strength is fixed to be ϵ_C–O_ = 0.040 kcal/mol while the lengths of water clusters are varied, and (c, d) the length of water clusters is fixed to be 60 Å while the values of ϵ_C–O_ are varied.

Using Verlet algorithm eqs 16 and 17, we plot the position *Z* and the velocity *V* of the water clusters moving inside a carbon nanotube of length 500 Å and radius *b*_1_=13.56 Å (Figure 5). First, we investigate the effect of the length of water cluster on *Z* and *V* as shown in Figure 5a,b. For these figures, we use ϵ_C–O_ = 0.040 kcal/mol and the corresponding *b*_2_ = *b*_2_^*^ = 10.40 Å, which maximizes the suction energy, hence giving rise to maximum velocity. The time for the water clusters to reach the other side of the channel is around 120 ps (Figure 5a). Because of its smallest mass, the cluster with the shortest length moves fastest and achieves the maximum velocity of 545 m/s at 5.5 ps (Figure 5b). As water clusters continue to travel along the channel, they experience friction force and thus, the velocity decreases to around 300 m/s when they reach the other side of the channel.

Next, we consider the effect of ϵ_C–O_ on the displacement and velocity of the water cluster moving along the carbon nanotube. In Figure 5c,d, the length of the water cluster is assumed to be 2*d* = 60 Å and the radius of the water cluster is taken to be *b*_2_ = *b*_2_^*^ = 10.40 Å. As shown in Figure 4b, larger interaction strength results in stronger interacting force and thus, we see in Figure 5c,d that water clusters with higher ϵ_C–O_ move faster at the start. However, a higher interaction strength ϵ_C–O_ between carbon nanotube and water molecules also implies strong binding between water and the tube’s wall, hence higher frictional forces. Thus, the clusters with lower ϵ_C–O_ travel faster in the long term (Figure 5c,d).

**Figure 6:**
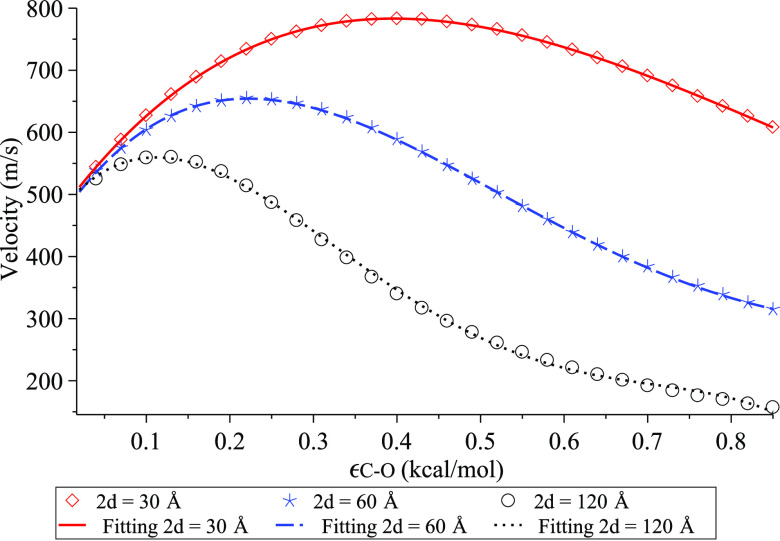
Maximum velocity for various water cluster lengths 2*d* and interaction strengths ϵ_C–O_.

In Figure 6, we plot the relation between the maximum velocity V* as a function of the interaction strength ϵ_C–O_ for each of the water cluster lengths 2*d* = 30, 60, and 120 Å. The figure confirms that the shorter the water cluster, the higher the maximum velocity. However, as the interaction strength ϵ_C–O_ increases, we see a decline in the maximum velocity for all sizes of the water clusters. This is expected because large values of ϵ_C–O_ imply a hydrophilic surface, which leads to strong binding between water molecules and the surface of the carbon nanotube, hence slowing down the transport of water clusters inside the nanotube. These findings agree with Falk et al.,^10^ Papadopoulou et al.,^11^ and Hummer et al.^12^

While the values of ϵ_C–O_ presented in previous studies are ∼0.04 to 0.16 kcal/mol,^11,16^ using the fourth-order polynomials to fit with numerical results with the least square error *R*^2^ = 0.99, we obtain the equations to describe the relationship between V* and any values of ϵ_C–O_ for each of the water cluster lengths 2*d* = 30, 60, and 120 Å, namely
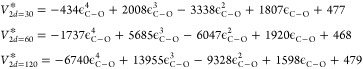
21Using eq 21 for each size of the water cluster, we also find the critical values of the interaction strength ϵ_C-O_^*^ and the corresponding maximum velocity V* as shown in Table 1. We comment that based on Rappé et al.,^16^ ϵ_C–O_ = 0.07937 kcal/mol and that in Papadopoulou et al.,^11^ the values of ϵ_C–O_ considered are from 0.04 to 0.16 kcal/mol. Thus, for ϵ_C–O_ ∈ [0.04,0.16], our results indicate that the maximum velocity can reach as high as 500–700 m/s.

**Table 1:** Critical Interaction Strength ϵ_C–O_^*^ and the Corresponding Maximum Velocity V* for Each of the Water Cluster Lengths

cluster length 2*d* (Å)	critical interaction strength ϵ_C–O_^*^ (kcal/mol)	maximum velocity V* (m/s)
30	0.396	783.143
60	0.222	654.200
120	0.111	559.512

For a larger size of carbon nanotubes, while results are not presented here, we comment that for any values of the radius of the carbon nanotube (*b*_1_) we can determine the corresponding optimum radius of water cluster (*b*_2_^*^) for any cluster lengths 2*d* and ϵ_C–O_. However, the velocities obtained for larger nanotubes are found to be less than those derived from using smaller nanotubes. This result is in agreement with Falk et al.^10^

## 5 Summary

Assuming no external force or pressure, this paper demonstrates ultrafast transport of a water cluster inside a carbon nanotube. In our model, a water cluster is assumed to be a cylinder comprising hydrogen (H) and oxygen (O) atoms with the ratio of H:O as 2:1. A carbon nanotube is modeled as a cylindrical channel comprising only carbon atoms. The interaction energy arising from the van der Waals forces between water clusters and carbon nanotubes is determined using the 6-12 Lennard-Jones potential. Adopting a continuum modeling approach, we obtain analytical expressions for the total interaction energy and force between two nested cylinders as a function of the dimensions of the two cylinders, their relative positions, and their atomic parameters. We then use the energy and force to study the effect of the size of the water clusters and the wettability inside the nanotubes on the dynamics behavior of water clusters inside carbon nanotubes.

Here, we assume three lengths of the water clusters which are 2*d* = 30, 60, and 120 Å. For the wettability, which is controlled by the interaction strength ϵ_C–O_, we examine its values in the range 0.04–0.16 kcal/mol. Furthermore, the cylindrical channel is assumed to be a (20, 20) carbon nanotube of radius 13.56 Å with a length of 500 Å. First, we determine the largest radius *b*_2_^°^ of the water cluster that can be encapsulated inside the channel (at zero suction energy) and the radius *b*_2_^*^, which corresponds to the maximum suction energy leading to the maximum velocity for the water cluster. We note that the intermolecular distance between a water cluster and a carbon nanotube is ∼2.7 to 3.1 Å regardless of the dimensions of water clusters and carbon nanotubes. However, this distance is slightly affected by ϵ_C–O_ since *b*_2_^°^ and *b*_2_^*^ slightly decrease when ϵ_C–O_ increases due to stronger interaction between water and carbon nanotube. We further observe a lower energy level inside the channel for a longer water cluster indicating a more stable structure. This is because there is more contact surface between the two molecules.

In terms of modeling dynamics of water clusters, Verlet algorithm is employed to determine the position and the velocity of the cluster in the carbon nanotube. The length of the water cluster significantly impacts its velocity. We find that the cluster with a shorter length reaches its maximum speed faster than the longer cluster. For the effect of the wettability on the speed of water clusters, we find for all cluster lengths that the higher the value of ϵ_C–O_, the lower the velocity of the water cluster.

Our findings in this paper demonstrate that carbon nanotubes can be used as nanopumps for ultrafast water transport and nanofiltration in drug delivery systems. By optimizing the physical parameters of water and carbon nanotube, the desired velocity can be achieved, which may be useful for the development of future ultrahigh-speed nanodevices.

## Figures and Tables

**Figure 7:**
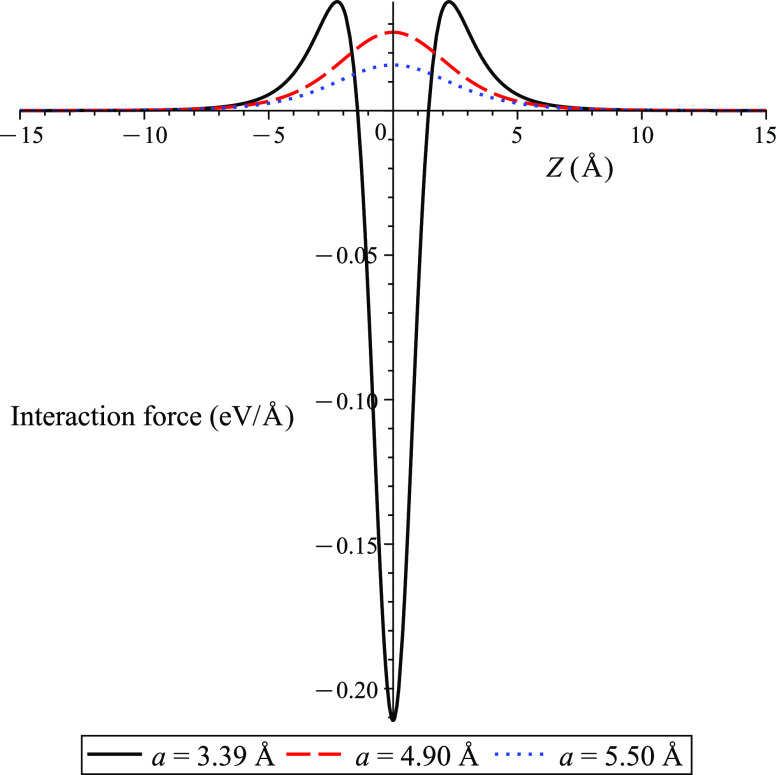
Interaction force for a spherical water molecule and a carbon nanotube of radius *a*.

## Appendix: Interaction between a Single Water Molecule and a Carbon Nanotube

Here, we present results using a continuum approach to model a single water molecule as a sphere inside a carbon nanotube. We also compare these results with those obtained from a hybrid discrete-continuous approach and molecular dynamics simulations^23,24^ to validate our assumption used in Section 2, where we consider a single water molecule as a sphere.

In Cox et al.,^14^ the interaction energy between a sphere and a cylindrical nanotube was determined using a continuum approach and the Lennard-Jones potential. Adopting the energy formulation in Cox et al.^14^ for a carbon nanotube and a sphere of a water molecule with radius 0.9109 Å (H–O bond length), we find that the minimum radius of the nanotube that will accept the sphere of a water molecule is 3.4648 Å, and the maximum suction energy occurs for a nanotube of radius 3.9407 Å. These two critical values of nanotube radii are in excellent agreement with results obtained from both hybrid discrete-continuous approach and molecular dynamics simulations.^23,24^ As an example, we plot in Figure 7 the force distribution for the spherical water molecule entering a carbon nanotube of three different radii. This figure is consistent with Figure 2 of Hilder and Hill^23^ and Figure 3 of Ansari and Kazemi.^24^

This agreement between the discrete atomistic and the continuum representations of a water molecule motivates and justifies the assumption of modeling a water molecule as a sphere, as shown in Section 2.
